# Postgraduate medical training in Germany: A narrative review

**DOI:** 10.3205/zma001570

**Published:** 2022-11-15

**Authors:** Elizabeth Sierocinski, Leonard Mathias, Julia Freyer Martins Pereira, Jean-François Chenot

**Affiliations:** 1 Greifswald University Medical Center, Institute for Community Medicine, Department of General Practice, Greifswald, Germany

**Keywords:** postgraduate medical training, postgraduate medical education, residency, specialty training, curriculum, regulations

## Abstract

The structure and content of the training phase following completion of medical school, referred to in most countries as postgraduate medical training, varies between countries. The purpose of this article is to give national and international readers an overview of the organisation and structure of postgraduate medical training in Germany.

The content and duration of postgraduate training in Germany are stipulated by state medical boards, officially termed associations (*Landesärztekammer*). In a periodically updated decree, the federal German medical association (*Bundesärztekammer*) provides a template for postgraduate medical training structure (*Musterweiterbildungsordnung*), which is adapted by the state medical associations. Admission to postgraduate medical training in Germany takes place by way of open, free-market selection. Based on the traditional assumption that junior doctors acquire all necessary clinical skills “on the job”, formal education in the form of seminars, lectures, or preorganised, detailed rotation plans through various specialties or wards is largely absent. Requirements for postgraduate medical training focus on the fulfilment of broad categories of rotations rather than specific content or gaining competencies. With few exceptions, no structured educational programs with curricular learning objectives exist. Limited funding impedes program development and expansion. Junior doctors bear the primary organisational responsibility in their training, which often results in extended training times and dissatisfaction. Structured training programs which prioritise skill-building and formal education are needed to support junior doctors and ensure their competence in primary and specialty care.

## Abstract (de)

Die strukturellen und inhaltlichen Aspekte der Facharztweiterbildung (Englisch: *postgraduate medical training*) sind in vielen Ländern verschieden. Dieser Artikel soll nationalen und internationalen Leser*innen einen Überblick über die Organisation und Struktur der Facharztweiterbildung in Deutschland geben.

Der Inhalt und die Dauer der Facharztweiterbildung werden durch die Landesärztekammern festgelegt. Die Weiterbildungsordnungen der Landesärztekammern orientieren sich an der von der Bundesärztekammer veröffentlichten Musterweiterbildungsordnung. Ärzt*innen in Weiterbildung in Deutschland bewerben sich auf offene Stellen im offenen, kompetitiven Arbeitsmarkt. Es wird weitgehend erwartet, dass die notwendigen Kompetenzen und Kenntnisse über die tägliche Arbeit im jeweiligen Fachgebiet erworben werden. Formale Weiterbildungsangebote im Sinne von Seminaren oder Vorlesungen sowie organisierte Rotationen durch verschiedene Weiterbildungsabschnitte werden selten oder nicht angeboten. Es gibt nur wenige strukturierte Weiterbildungsprogramme und wenig Förderung hierfür, sodass die Ärztin/der Arzt in Weiterbildung selbst für die Erfüllung der Anforderungen der Weiterbildungsordnung verantwortlich ist. Dies führt häufig zu verlängerten Weiterbildungszeiten und Unzufriedenheit mit der Weiterbildung. Strukturierte Weiterbildungsprogramme und eine Professionalisierung der Weiterbildung müssen in Deutschland erst noch etabliert werden, um Ärzt*innen in Weiterbildung beim gesicherten Kompetenzerwerb zu unterstützen.

## 1. Introduction

Ensuring clinical competence of doctors within their specialty is a major public health issue that highlights the importance of effective medical training. The phase following medical school in Germany has been compared to other countries [1], [2] but has not been described in detail in international publications since 1997 [3]. Postgraduate medical training in Germany has undergone several changes since then [4], [5]. Undergraduate medical education has previously been described in detail [6]. 

About 10,000 medical students graduate in Germany each year [7] and over 95% plan to begin postgraduate medical training [8]. Historically, medical education in Germany has enjoyed an excellent reputation [9]; however, over 70% of surveyed medical students have reported considering training outside of Germany and many German-trained physicians emigrate to other countries every year [8], [10], [11]. This may be partly explained by perceived better working conditions or a better salary e.g. in the USA, Scandinavian countries, or Switzerland, as well as structured postgraduate medical education programs [12]. Germany and other European countries have been experiencing shortages in the medical workforce, especially in rural areas, for years [13], [14]. The improvement of training conditions for junior doctors is cited among potential solutions to this problem [15].

This narrative review provides national and international readers an overview of the organisation, structure, and current challenges of postgraduate medical training (*Facharztweiterbildung)* in Germany. 

### Terminology and methods

Table 1 (Tab. 1) provides an overview of key English terms used. Most countries refer to the phase after medical school as postgraduate [1], [https://www.acgme.org/about-us/overview/]; trainees or residents are called junior doctors in this text and others [1]. Attachment 1 provides an overview of German terms.

#### Education vs. training

The World Federation for Medical Education (WFME) defines postgraduate medical education as “the phase of medical education in which doctors develop competencies under supervision after completion of their basic medical qualification” taking place within structured programs with clearly defined didactic elements [16]. Postgraduate medical training is considered the practical aspect of postgraduate medical education. To emphasise the dominance of practical, “on-the-job” postgraduate learning in Germany, we use the term training in this text.

##### Search strategy

We completed a selective literature search for articles published at any date regarding specialist training, residency and (post)graduate medical education/training in Germany. Data from international journals, official organisations, German medical journals not indexed in PubMed, and testimonials of personal experiences in the field were also included in our narrative synthesis. 

## 2. Structure and organisation

### 2.1. Prerequisites and application process

Table 2 (Tab. 2) provides an overview of the key aspects of training in Germany. First, a license to practice medicine (*Approbation*) is needed to begin postgraduate medical training. License registration is regulated by the responsible Ministry (*Bundesministerium für Gesundheit*) [https://www.gesetze-im-internet.de/b_o/BJNR018570961.html]. Medical school graduates in Germany typically apply for and receive their license weeks after graduation. 

Special regulations apply for junior doctors who completed undergraduate training outside the European Union, which are increasingly represented in the workforce [17], [18], [19]. The process is often fraught with bureaucratic hurdles and other difficulties [20]. Specific prerequisites are determined on a state level and are managed by the respective State Examination Office (Landesprüfungsamt) or the designated office. Medical graduates from the EU have an easier path to licensure due to recognition treaties [21].

Junior doctors with a German medical license can apply for vacant posts in hospitals or practices. This open, free-market selection is unlike countries such as UK and the United States: First, there is no central application or matching process; second, training can be started or interrupted year-round; third, contracts are often short-term and do not necessarily fully cover postgraduate training [1]. The demand for junior doctors exceeds the supply, consistent with the current physician shortage [22], [23], [24].

Employment opportunities exist for physicians without completed specialty training in industry, research, administration, or clinical work. However, completion of postgraduate training and board certification as a specialist (*Facharzt/-ärztin*) is an important career step and considered highly desirable.

#### 2.2. Regulations and legal framework

Postgraduate specialty training is regulated by 17 regional (state) medical associations (*Landesärztekammer*) [25] and is loosely based on recommendations of the German Federal Medical Association (*Bundesärztekammer*) [26]. State medical associations are responsible for the accreditation of training sites for physicians in training, acceptance of completed rotations as reported by junior doctors, appraising applications from candidates presenting for board certification and administering board examinations (*Facharztprüfung*). 

#### 2.3. Registration as a junior doctor

There is no systematic, nation-wide registration of junior doctors in Germany. Thus, the number of junior doctors can only be estimated. According to a report of the German Federal Medical Association, of the 409,121 doctors working in Germany in 2020, approximately one-third or 117,699 had not yet completed specialty training [23]. This number only roughly approximates the number of junior doctors. Given the annual number of medical school graduates and the assumption that postgraduate medical training in Germany can be completed within five to six years, this number of junior doctors is unexpectedly high.

In contrast, the number of specialties awarded is reliably collected. In 2020, 13,861 board certifications were awarded; of these, 1,666 were in General Practice [23].

#### 2.4. Medical specialties

Attachment 2 provides an overview of specialties recognised by the German Federal Medical Association. Subspecialties (*Schwerpunkte*), such as gynaecological oncology, and additional qualifications (*Zusatzbezeichnungen*), such as palliative care and emergency medicine, are possible; these approximate the concept of fellowship in North America (see attachment 3 ) and can be completed parallel to or after postgraduate medical training. 

#### 2.5. Training sites and trainers

Postgraduate medical training is not formally attached to academic medical centres and is provided largely outside of organised programs [27]. Training positions are offered by hospitals as well as private practices. Board-certified specialists can apply to obtain a license to supervise professional training (*Weiterbildungsbefugnis*) in their specialty. Prerequisites are generally several (typically three) years of working experience, sufficient patient flow and structural conditions such as quality assurance protocols and provision of specific medical services. The decision to grant this license is mainly based on the documentation submitted to the state medical association (*Landesärztekammer*); onsite visits or personal interviews are rare. Teaching qualifications or experience are not required. In contrast, knowledge and application of educational/learning theories are required in the Netherlands [27] and at least three years of educational experience are required for certification to supervise postgraduate medical training in the United States [28]. 

Supervisors have limited formal duties concerning the training advancement of junior doctors. Hospitals and supervisors are not required to provide formal bedside teaching, lectures or case discussions; as such, these are limited or absent, and junior doctors must integrate learning needs into routine work and take overtime to learn skills [29], [30]. This exemplifies traditional “on the job” postgraduate training, which has been criticised for the lack of value it places on high-quality, thorough training [29], [31]. 

After granting permission to supervise postgraduate training and registering supervisors in online catalogues [32], state medical associations have no obligation (aside from their general duty to regulate training) to conduct any form of monitoring or evaluation of supervisors and training facilities.

#### 2.6. Training framework

Mandatory rotations for each specialty are recommended by a template for postgraduate medical training (*Musterweiterbildungsordnung*) published by the German Federal Medical Association (*Bundesärztekammer*) [33]. State medical associations generally adhere to the template [34]. Table 3 (Tab. 3) illustrates regional differences with the example of General Practice.

Although the most recent update to the template for postgraduate medical training represents a slight shift from a time- and number-based system to a competency-based one, curricular goals remain largely absent from formal requirements. The documentation of required numbers of procedures without competency assessment (*Richtzahlen*) continues to play a significant role. This is contrary to recommendations of the WFME and programs in countries such as the United States, in which the acquisition of competencies forms the cornerstone of postgraduate curricula [16], [28].

Additional courses offered by third parties such as radiation safety are required or, in the case of ultrasound, may be highly useful to junior doctors to gain skills. The burden of costs and invested time often fall upon the junior doctors, so that courses are attended on weekends, after work or during vacation. 

The fulfilment of training requirements is the responsibility of the junior doctors. Each rotation is organised by applying for the respective position. Highly desired and/or specialised rotations often have waiting times due to limited availability. As a result, training may take longer than expected. For example, only 45% of junior doctors in surgery manage to complete training requirements within the predetermined six years [35]. Training in general practice should take five years but requires a mean of 9.5 years [36]. Delays of at least a year are typical in most disciplines [37], although taking a leave of absence between working positions or switching to part-time are possible and may be due to diverse factors including taking parental leave or simply desiring a break between rotations. Longer training times than expected are associated with decreased long-term vocational satisfaction on the part of junior doctors and low ratings of training [38].

There are no interim examinations and no mandatory evaluations of training progress prior to board examination. In contrast, the United States and the United Kingdom require standardised testing during postgraduate medical training (e.g., in-training exams and core training exams, respectively). 

#### 2.7. Board certification

A junior doctor who has fulfilled training requirements is eligible to register for licensing examination (*Facharztprüfung*). Certificates of rotation completion (*Weiterbildungszeugnis*) and approval of entries of procedures and rotation times in a logbook (*Logbuch*) must be obtained from supervisors and submitted for assessment to the state medical association [33]. This may result in unexpected hardship for junior doctors, as certain rotations or procedures may fail to be certified by supervisors or accepted by state medical associations. This can result from misinformation from the supervisor/training site or misunderstandings on the part of the junior doctor about which rotations count for a specific specialty. In a worst-case scenario, a junior doctor attempting to sign up for board examination may find out that months to years of training must be repeated. 

Once completeness is established, candidates are admitted for the individual oral licensing examination lasting roughly 30 minutes. The examination committee consists of at least three medical specialists, two of whom must be specialists in the candidate’s aspired field. No specific qualification or preparation is required for board examiners; this is in contrast to medical schools which require examiners to take a preparatory course. After passing board examination, the title of board-certified specialist is conferred.

Board certification is a prerequisite to work independently or to apply for registration at the Association of Statutory Health Insurance Physicians (*Kassenärztliche Vereinigung*), which in turn is a prerequisite for billing privileges for patients with statutory health insurance. 

**Figure 1:**
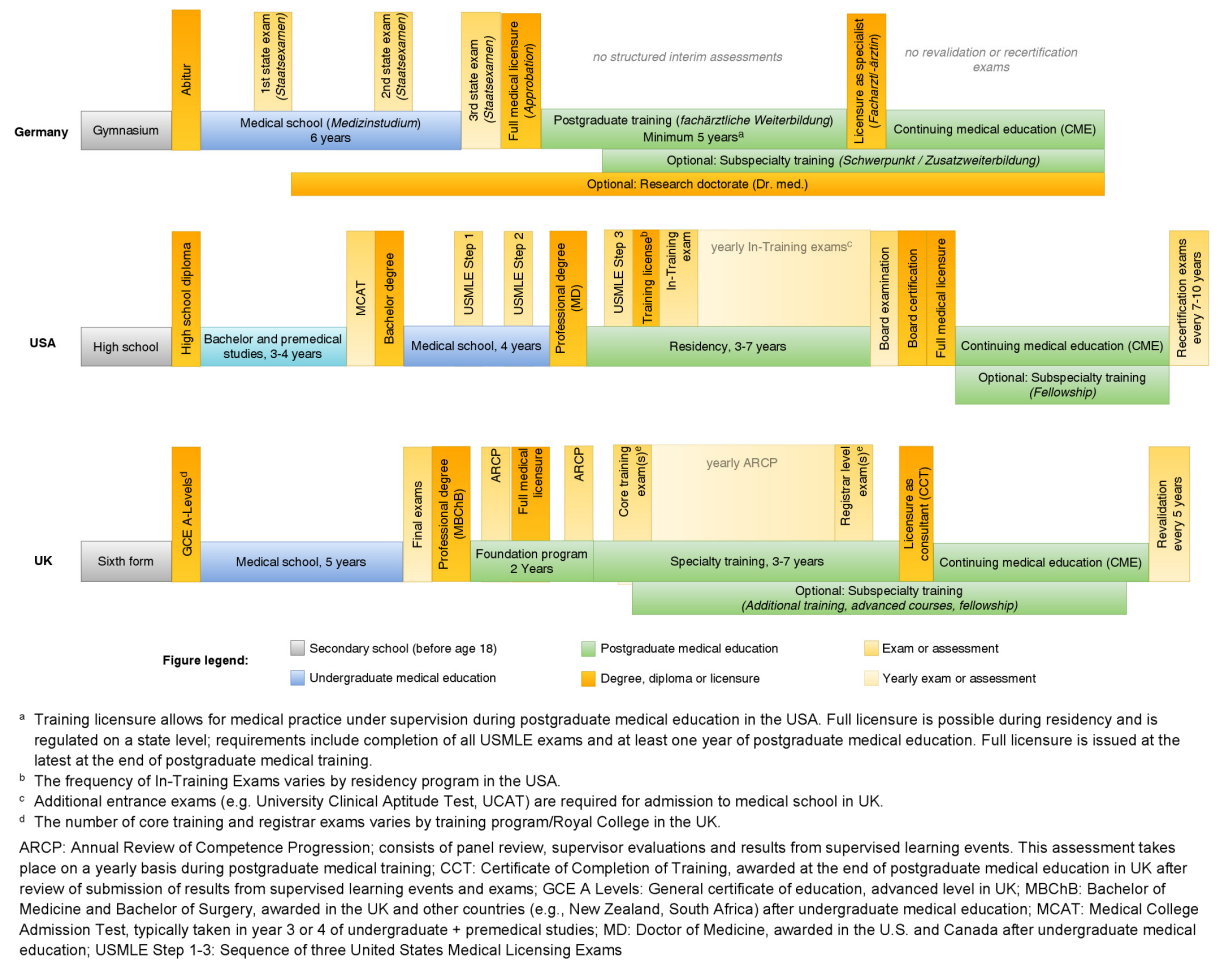
Comparative overview of medical training and milestones in Germany, United States and United Kingdom

Figure 1 (Fig. 1) provides a comparative overview of the structure and milestones of medical education in Germany, the United States and the United Kingdom. Detailed comparisons between these and other countries have been published elsewhere [1], [2]. 

#### 2.8. Salary and funding

The salaries of junior doctors in Germany are relatively high when compared to other professions in Germany and to salaries of junior doctors in other countries such as UK, France, the Netherlands and the United States. However, lack of sufficient funding for medical training, consisting of salary and coverage of education resources and needs, is a long-standing problem in Germany and other European countries [39]. Although there have been important reforms in the economic support of junior doctors in recent years [40], these have been criticised as insufficient. It has been argued that a too-small educational subsidy included in the Disease-Related-Groups (DRG) model for the financing of inpatient care precludes the prioritisation of postgraduate medical training [41]. For example, in surgical specialties, junior doctors are hindered in acquiring operating skills because they are more frequently implemented on the wards than in the operating room [42]. This is thought to be partly due to the prioritisation of cost-/time-effective use of operating rooms over cost-/time-intensive training [42].

Regarding outpatient training, external funding was increased in 2019 to support 7,500 outpatient training posts in general practice and 2,000 in other specialties [43]. In the context of the estimated over 100,000 junior doctors in 2020, this is an insufficient number of ambulatory training posts. As a result, ambulatory rotations in many specialties are difficult to obtain and most junior doctors are employed and taught in inpatient settings [44].

#### 2.9. Ambulatory training

The majority of medical care in industrialised countries is delivered in an ambulatory setting and roughly half of the physician work force in Germany is in the ambulatory sector. Outpatient postgraduate training is mandatory in many countries and is a basic WFME standard [16]. In Germany, ambulatory training is required for General Practice, but not in many specialties that frequently practiced in an ambulatory setting, such as Gynaecology, Paediatrics and Rheumatology. Due to this and the aforementioned mismatch in number of ambulatory training positions, it is possible for a doctor to become an ambulatory gynaecologist after board examination, although he or she has exclusively trained in hospitals with no experience in preventive services or ambulatory antenatal care.

#### 2.10. Evaluation of training

There is no transparent, systematic evaluation of postgraduate medical training in Germany, although this has been demanded [45]. The last national evaluation was performed in 2011 [46]. That junior doctors are not typically offered the opportunity to evaluate training sites during or after rotations has been criticised repeatedly [4], [31]. However, regional exceptions exist: the Medical Association of Westfalen-Lippe performs an evaluation every 2 years [47]; Thuringia, Berlin and North-Rhine have also begun evaluations [48], [49]. As opposed to other evaluations, these results are public, potentially enabling junior doctors to find well-evaluated training positions. Evaluations of certain elements of postgraduate medical training are now required in General Practice, but these do not include assessments of individual rotation sites [43]. 

#### 2.11. Regulation and planning of the physician workforce 

There is no formal regulation or planning of the number of training positions in any specialty. The number of positions solely reflects the staffing needs of hospitals and private practices, especially for night shifts and weekends. In contrast, the number of board-certified outpatient doctors with billing privileges with the statutory health insurance is regulated by the State Associations of Statutory Health Insurance Physicians.

 A shortage of junior doctors and board-certified doctors exists in virtually all specialties since the early 2000s. An exception is Neurosurgery, in which the number of specialists has been projected to exceed national demand [50]. To balance cases of over- and undersupply, workforce planning and regulation in postgraduate training has been demanded [50]. In rural areas, attempts are being made to increase the attractiveness of unfilled training positions. For example, multiple states now reserve a percentage of medical school spots for students who pledge to work in an underserved rural area for at least 10 years after graduation [51]. 

## 3. Areas of improvement

### Teaching quality 

The dissatisfaction of junior doctors is rooted to a large extent in the lack of feedback, mentoring and formal instruction during work hours [52], [53]. Structured educational opportunities and training requirements for supervisors are recommended by the WFME and the German Society for Medical Education (GMA) and may contribute to the improvement of postgraduate medical training in Germany [16], [54]. Workday “de-cluttering” via delegation of appropriate tasks to adequately trained nurses or physician assistants and an increase in the relative weights of DRGs for teaching hospitals have also been suggested [42], [54].

#### Data gathering

Formal registration of junior doctors by specialty can help identify strengths and weaknesses of the system, e.g., how long junior doctors take to complete requirements for postgraduate medical training.

#### Quality control

Evaluations of supervisors, training sites, and the competencies of junior doctors identify areas for improvement [55]. Other countries implement re-accreditation as a method of quality control in which training programs are evaluated (on-site) for structural and educational requirements [16], [28]. This has been discussed in Germany and criticised on the one hand for the significant costs in terms of time and personnel required [31]. On the other hand, regular external evaluations may improve training quality [54], [55]. 

#### Competency-based requirements and assessments

A competency-based medical education focusing on the honing of the actual abilities of the junior doctor [56] would require de-emphasis of time- and workplace-based training in Germany and the development of new ways to assess the progress of training. Competency-based Entrustable Professional Activities (EPAs) have already been suggested for medical education in Germany [57], [58]; in particular, EPAs for postgraduate training in paediatric primary care have been developed [59]. 

## 4. Recent developments

### Funding

Joint funding from the state statutory health board and statutory/private health insurance providers for regional centres for postgraduate medical training in General Practice (*Kompetenzzentren Weiterbildung*) was written into law in 2015 [40]. Seminars, workshops and lectures are offered at regular intervals at no or low additional cost to junior doctors and have been well-received [42].

#### Postgraduate training networks

To guarantee the completion of all training requirements for General Practice within the recommended time frame, postgraduate training networks (*Weiterbildungsverbund*) offer long-term contracts with a clear inpatient and outpatient rotation plan. Initial evaluations show high levels of satisfaction and the number of interested applicants is increasing [60]. The availability of these networks varies by region and networks for other specialties are uncommon.

#### Train-the-trainer seminars

Structured, interactive train-the-trainer seminars have been developed to improve the didactic competencies of specialists who are licensed to supervise postgraduate medical training in Germany. These have been positively received by participants [61]. These seminars now form a mandatory part of the licensure process for trainers in lower saxony [62]; however, they are not available or required in all German states.

#### Shift toward competency-based requirements

The 2018 update to the federal template showed a shift towards competency-based requirements, particularly in general practice [4], [5]. However, number-based requirements in numerous specialties remain and competency-based assessments have not yet been established.

## 5. Conclusion

It is possible to receive good postgraduate medical training in Germany. However, postgraduate medical training in Germany is complex and widely conducted as though it were a by-product of patient care. There is no structured transfer of knowledge and no intermittent verification of theoretical and practical milestones. Although the training content is subject to elaborate administrative regulations, a lack of financing prevents the development of professional structures to ensure the quality of postgraduate medical training. There is increasing awareness that continuous evaluation, Train-the-trainer qualifications, structured training, and supervision of junior doctors are urgently needed. This will require cooperation between state medical associations charged with administrative processes, institutions involved in health care financing and medical training facilities. We cannot afford to pay the price for poor medical care delivered by inadequately trained physicians, or to lose physicians from the workforce due to frustrating training conditions, instead of investing funds for the development and maintenance of high-quality postgraduate medical education in Germany.

## Authors

The authors ES and LM contributed equally to the article.

ES completed undergraduate medical training in the United States; LM completed undergraduate medical education in Germany; JFMP completed undergraduate and part of postgraduate medical training in the United Kingdom; ES, LM, JFMP are currently junior doctors in Germany; JFC completed postgraduate medical training in the United States.

JFC was chair of the section postgraduate medical training of the German College of Family Medicine (DEGAM) and chief representative of General Practitioners in the negotiations for the 2018 German Federal Medical Association template for postgraduate medical training. None of the opinions expressed here reflect official positions of these organisations.

## Competing interests

The authors declare that they have no competing interests. 

## Supplementary Material

Glossary of terms

Recognised medical specialties in Germany as per the federal template for postgraduate medical training

Subspecialties and additional qualifications as per the federal template for postgraduate medical training

## Figures and Tables

**Table 1:**
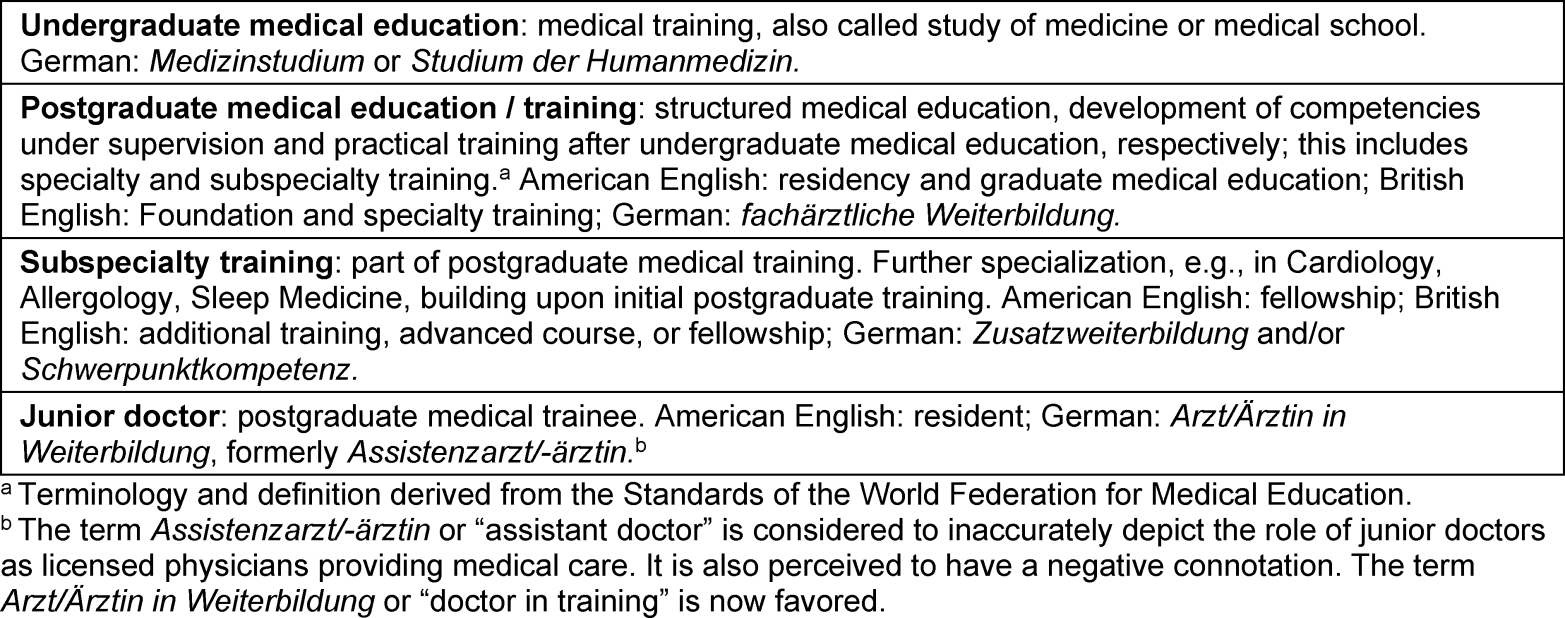
Terminology

**Table 2:**
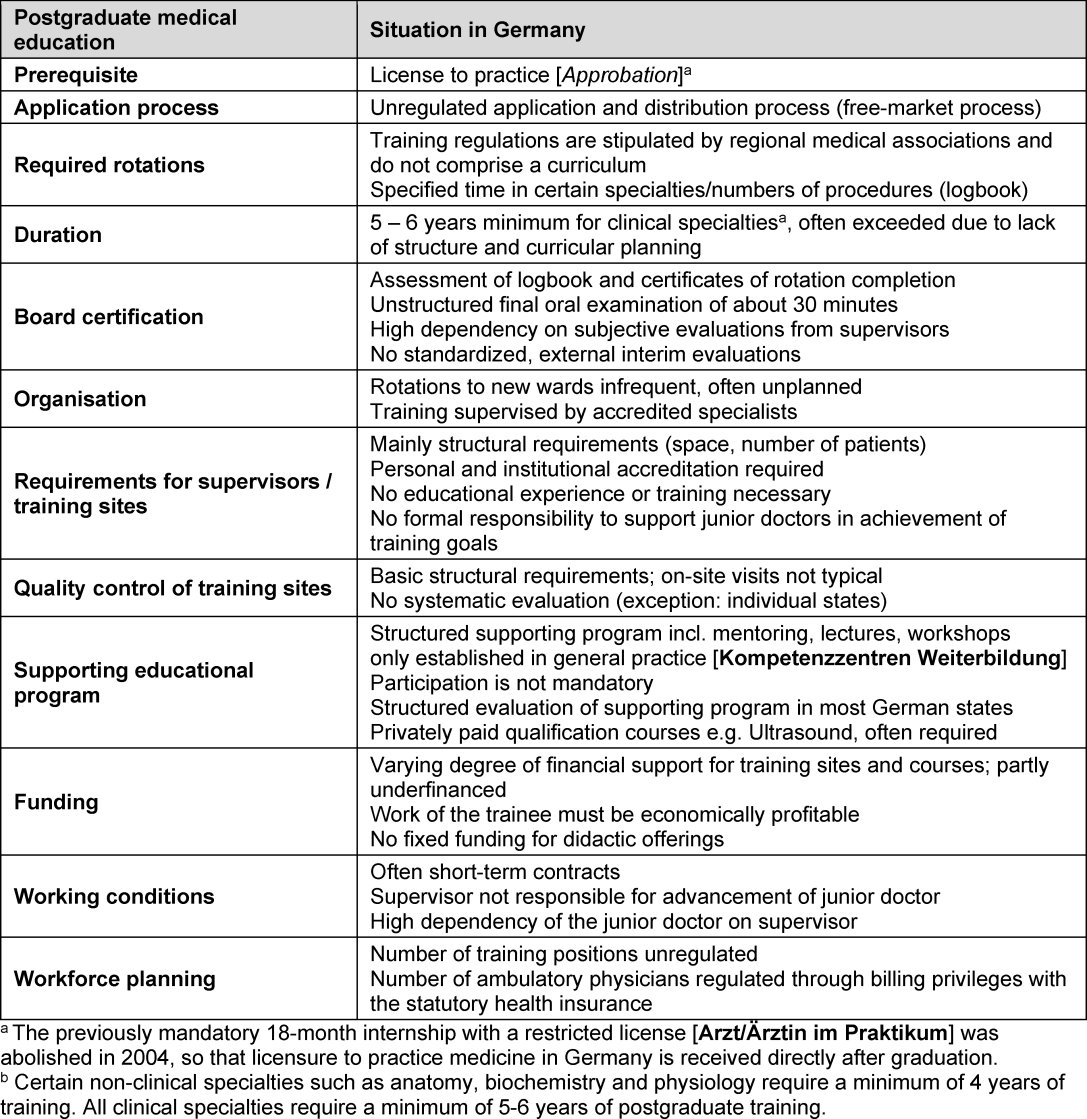
Postgraduate medical education in Germany in brief

**Table 3:**
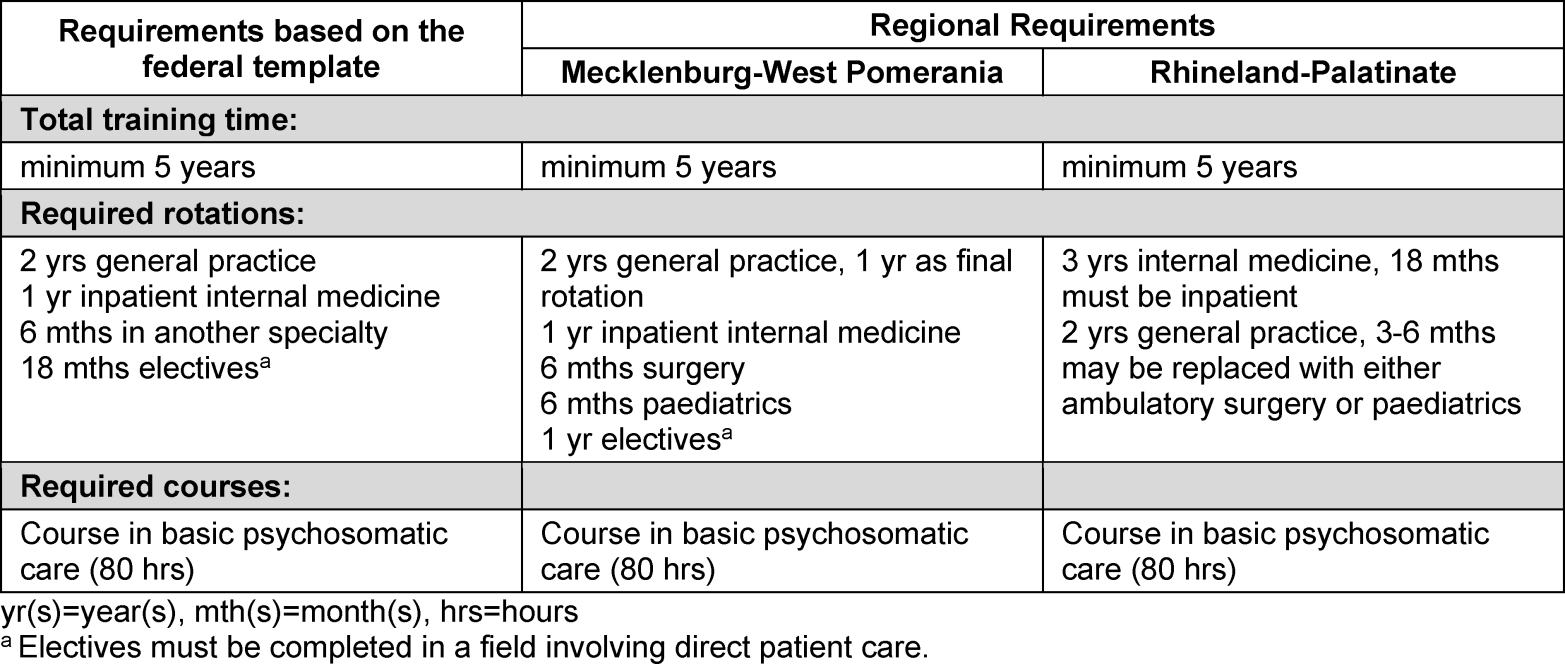
Differences in regional rotation requirements for postgraduate medical training in Germany based on the example of general practice/family medicine
