# Supplementary material for: Postgraduate medical training in Germany: A narrative review
Source: GMS J Med Educ. 2022 Nov 15;39(5):Doc49. doi: 10.3205/zma001570 (PMC9733474; doi:10.3205/zma001570)
Supplement: Glossary of terms [file JME-39-49-s-001.pdf]

Attachment 1: Glossary of terms

When available, translations suggested by the institution itself or the term suggested on the webpage of the federal statutory health board (https://www.kbv.de/html/dictionary.php) were utilized. To prevent misunderstandings, we provided the correct German term in brackets when using a term for the first time.

For translations of specialties such as internal medicine or surgery, please see **Attachment 2**.

Arzt/Ärztin im Praktikum*:* 18-month internship with a provisional medical license, completed after completion of undergraduate medical education and prior to beginning postgraduate medical training. Abolished in 2004.

Bundesärztekammer: German federal medical board, officially termed the German federal medical association. It is the federal working group of the state medical associations (*Landesärtzekammer*).

Diplom-Mediziner/-in (Dipl. Med.): professional title conferred to medical school graduates in German Democratic Republic prior to German reunification in 1990. East German physicians who graduated prior to 1990 retain the title.

Facharzt/-ärztin: Board-certified physician; requirement is completion of postgraduate medical training and licensing examination in a given specialty.

Facharztprüfung: licensing / board exam completed at the completion of postgraduate medical training in a given specialty; requirement for board certification in that specialty. Carried out by the respective state medical association (*Landesärztekammer*).

Facharztweiterbildung: postgraduate medical training, other terms include residency or graduate medical education (American English) and foundation / specialty training (British English).

gesetzliche Krankenkassen: statutory health insurance providers.

Kassenärztliche Vereinigung: regional (state) statutory health board. The statutory health boards work as bargaining partners between the statutory health insurances (**gesetzliche Krankenkassen**) and the doctors. Funds for out-patient care are collected and distributed among doctors according to factors such as the number of patients seen, procedures performed. In the vast majority of cases, physicians who wish to treat and bill patients who are covered by the statutory health insurance must be registered with the statutory health board.

Kompetenzzentren Weiterbildung: regional centers for postgraduate medical education which serve as supporting educational programs, jointly funded by the state statutory health board and statutory and private health insurance providers. These centers offer mentoring programs as well as regular lectures and workshops. Currently only available for postgraduate medical education in General Practice (and Pediatrics in some States, e.g. Hessia).

Landesärztekammer: official translation: state medical associations; these are public/statutory bodies or medical boards responsible for the self-administration of physicians, with tasks including but not limited to accreditation of training sites for postgraduate medical training, verifying training progress reports submitted by physicians in training, appraising candidates presenting for board certification and for administering board examinations (**Facharztprüfung**) in each specialty. This institution also grants licenses to supervise postgraduate medical training (**Weiterbildungsbefugnis**) to board-certified physicians who apply and fulfill the qualifications.

Attachment 1 to Sierocinski E, Mathias L, Freyer Martins Pereira J, Chenot JF. *Postgraduate medical training in Germany: A narrative review*. GMS J Med Educ. 2022;39(5):Doc49. DOI: 10.3205/zma001570

Landesprüfungsamt: State Examination Office; part of the State Ministry responsible for Health in the respective State.

Logbuch: Logbooks in which junior doctors document completion of mandatory rotations and required numbers of procedures during postgraduate medical training. In the latest reform of the federal template in 2018, an online logbook was introduced to replace the previous paper format, which was meant to simplify and facilitate the transfer of logged activities between state medical associations.

Ministerium für Gesundheit (unterschiedliche Namen je nach Bundesland): State Ministry of Health (differently named in each federal state). Separate from the state medical associations (**Landesärztekammer**), this political institution (ministry) is responsible for issuing licenses to practice medicine to medical school graduates prior to beginning postgraduate medical training and verifying foreign medical graduates for licensure, as well as political oversight over the health sector in general.

Musterweiterbildungsordnung (MWBO): template for postgraduate medical training structure provided by the German federal medical association; this template is adapted by state medical associations to form requirements for residency training.

Praktischer Arzt/Praktische Ärztin: directly translated as “practical doctor”, this designation refers to a medical school graduate with at least 6 months of postgraduate medical training but without board certification in a specialty who, prior to 1992, opened a practice for general medicine and registered as a general physician with the regional statutory health board. This designation has been out of use since 1992, at which time board certification became a requirement to register with the statutory health board as a general practitioner.

Richtzahlen*:* minimum number of procedures or activities (e.g., EKG-reading, ultrasound examinations, specific surgical procedures) that must be logged during postgraduate medical training. These numbers should in general be fulfilled in order to be accepted for board examination by the state medical association. The required numbers lack an evidence-base and often exceed what can reasonably be seen and done in a hospital or practice.

Schwerpunkt: subspecialty, such as gastroenterology, endocrinology, and vascular surgery. Alongside additional qualifications (*Zusatzbezeichnungen*), subspecialty training approximates the US-American concepts of fellowships.

Weiterbildungsbefugnis: license to supervise postgraduate medical training or subspecialty training in a given field. Board-certified physicians with the requisite work experience, patient flow, and resources can apply.

Weiterbildungsverbund: postgraduate training network which coordinates contracts for junior doctors in General Practice, ensuring that all inpatient and outpatient rotations can be completed within the recommended 5 years. Currently available in a limited capacity.

Zusatzbezeichnung/Zusatzweiterbildung: Additional qualification that may be completed parallel to or after postgraduate medical training in a given specialty. Examples include palliative care, emergency medicine and sport medicine. Alongside German subspecialty (**Schwerpunkt**) training, these additional qualifications approximate the US-American and UK concepts of fellowships.
