# Supplementary material for: Postgraduate medical training in Germany: A narrative review
Source: GMS J Med Educ. 2022 Nov 15;39(5):Doc49. doi: 10.3205/zma001570 (PMC9733474; doi:10.3205/zma001570)
Supplement: Recognised medical specialties in Germany as per the federal template for postgraduate medical training [file JME-39-49-s-002.pdf]

Attachment 2*:* Recognised medical specialties in Germany as per the federal template for postgraduate medical training

**Specialty (German)**	**English translation**
Allgemeinmedizin[a]	General Practice
Anästhesiologie	Anaesthesia
Anatomie[b]	Anatomy
Arbeitsmedizin	Occupational medicine
Augenheilkunde	Ophthalmology
Biochemie	Biochemistry
Allgemeinchirurgie	General surgery
Gefäßchirurgie	Vascular surgery
Herzchirurgie	Cardiac surgery
Kinder- und Jugendchirurgie	Paediatric surgery
Orthopädie und Unfallchirurgie	Trauma and orthopaedic surgery
Plastische, Rekonstruktive und Ästhetische Chirurgie	Plastic and aesthetic surgery
Thoraxchirurgie	Thoracic surgery
Viszeralchirurgie	Visceral surgery
Frauenheilkunde und Geburtshilfe	Obstetrics and Gynaecology
Hals-Nasen-Ohrenheilkunde	Ear-nose-throat (ENT)
Haut- und Geschlechtskrankheiten	Dermatology and venereal diseases
Humangenetik	Human genetics
Hygiene und Umweltmedizin	Hygiene and environmental medicine
Innere Medizin	Internal medicine
Kinder- und Jugendmedizin	Paediatrics
Kinder- und Jugendpsychiatrie und -psychotherapie	Paediatric and adolescent psychiatry and psychotherapy
Laboratoriumsmedizin	Laboratory medicine
Mikrobiologie, Virologie und Infektionsepidemiologie	Microbiology, virology and infectious disease epidemiology
Mund-Kiefer-Gesichtschirurgie	Oral and maxillofacial surgery
Neurochirurgie	Neurosurgery
Neurologie	Neurology
Nuklearmedizin	Nuclear medicine
Öffentliches Gesundheitswesen	Public Health Services
Neuropathologie	Neuropathology
Pathologie	Pathology
Klinische Pharmakologie[b]	Clinical pharmacology
Pharmakologie und Toxikologie	Pharmacology and toxicology
Phoniatrie und Pädaudiologie	Disorders of speech, voice, and hearing in childhood
Physikalische und Rehabilitative Medizin	Physical and rehabilitative medicine
Physiologie[b]	Physiology
Psychiatrie und Psychotherapie	Psychiatry and psychotherapy
Psychosomatische Medizin und Psychotherapie	Psychosomatic medicine and psychotherapy
Radiologie	Radiology
Rechtsmedizin	Forensic medicine
Strahlentherapie	Radiotherapy
Transfusionsmedizin	Transfusion medicine
Urologie	Urology

[a]A special historical case is presented by the specialty of General Practice [**Allgemeinmedizin**], which has been a recognised specialty in Germany since 1968. Until 1992, the completion of postgraduate medical training in general practice was not a prerequisite to open a practice as a general practitioner and register with the statutory health board: a doctor with 6 months of working experience was allowed to work as a general practitioner under the label “practical doctor” [**Praktischer Arzt/Ärztin**]. Historical differences also existed between the German Democratic Republic and the Federal Democratic Republic: East German doctors received a professional title [**Diplom-Mediziner/-Medizinerin/Dipl. Med.**] automatically after graduation from medical school. These differences are still visible when visiting former East German states, as physicians kept their titles in the unified German medical system.
[b]Specialties such as anatomy, physiology or clinical pharmacology are research/teaching-focused and have no direct clinical connection.

Attachment 2 to Sierocinski E, Mathias L, Freyer Martins Pereira J, Chenot JF. *Postgraduate medical training in Germany: A narrative review*. GMS J Med Educ. 2022;39(5):Doc49. DOI: 10.3205/zma001570
