# Supplementary material for: Postgraduate medical training in Germany: A narrative review
Source: GMS J Med Educ. 2022 Nov 15;39(5):Doc49. doi: 10.3205/zma001570 (PMC9733474; doi:10.3205/zma001570)
Supplement: Subspecialties and additional qualifications as per the federal template for postgraduate medical training [file JME-39-49-s-003.pdf]

Attachment 3: Subspecialties and additional qualifications as per the federal template for postgraduate medical training

**Subspecialties (by field)**	**English translation**
Frauenheilkunde und Geburtshilfe<br>- Gynäkologische Endokrinologie und Reproduktionsmedizin<br>- Gynäkologische Onkologie<br>- Spezielle Geburtshilfe und Perinatalmedizin	Obstetrics and Gynaecology<br>- Gynaecological endocrinology and reproductive medicine<br>- Gynaecological oncology<br>- Special obstetrics and perinatal medicine
Innere Medizin<br>- Angiologie<br>- Endokrinologie und Diabetologie<br>- Gastroenterologie<br>- Hämatologie und Onkologie<br>- Infektiologie<br>- Kardiologie<br>- Nephrologie<br>- Pneumologie<br>- Rheumatologie	Internal medicine<br>- Angiology<br>- Endocrinology and diabetology<br>- Gastroenterology<br>- Haematology and Oncology<br>- Infectious diseases<br>- Cardiology<br>- Nephrology<br>- Pulmonology<br>- Rheumatology
Kinder- und Jugendmedizin	Paediatrics
- Kinder- und Jugend-Hämatologie und -Onkologie<br>- Kinder- und Jugend-Kardiologie<br>- Neonatologie<br>- Neuropädiatrie	- Child and adolescent haematology and oncology<br>- Child and adolescent cardiology<br>- Neonatology<br>- Paediatric neurology
Psychiatrie und Psychotherapie<br>- Forensische Psychiatrie	Psychiatry and psychotherapy<br>- Forensic psychiatry
Radiologie<br>- Kinder- und Jugendradiologie<br>- Neuroradiologie	Radiology<br>- Paediatric and adolescent radiology<br>- Neuroradiology
**Additional qualifications**	**English translation**
Ärztliches Qualitätsmanagement	Medical quality management
Akupunktur	Acupuncture
Allergologie	Allergology
Andrologie	Andrology
Balneologie und Medizinische Klimatologie	Balneotherapy
Betriebsmedizin	Occupational medicine
Dermatopathologie	Dermatopathology
Diabetologie	Diabetology
Ernährungsmedizin	Nutrition medicine
Flugmedizin	Aviation medicine
Geriatrie	Geriatrics
Gynäkologische Exfoliativ-Zytologie	Exfoliative gynecological cytology
Hämostaseologie	Haemostaseology
Handchirurgie	Hand surgery
Homöopathie	Homeopathy
Immunologie	Immunology
Infektiologie	Infectious diseases
Intensivmedizin	Intensive care
Kardiale Magnetresonanztomographie	Cardiac magnetic resonance imaging
Kinder- und Jugend-Endokrinologie und -Diabetologie	Child and adolescent endocrinology and diabetology
Kinder- und Jugend-Gastroenterologie	Child and adolescent gastroenterology
Kinder- und Jugend-Nephrologie	Child and adolescent nephrology

Attachment 3 to Sierocinski E, Mathias L, Freyer Martins Pereira J, Chenot JF. *Postgraduate medical training in Germany: A narrative review*. GMS J Med Educ. 2022;39(5):Doc49. DOI: 10.3205/zma001570

Kinder- und Jugend-Orthopädie	Child and adolescent orthopedics
Kinder- und Jugend-Pneumologie	Child and adolescent pneumology
Kinder- und Jugend-Rheumatologie	Child and adolescent rheumatology
Klinische Akut- und Notfallmedizin	Clinical acute and emergency medicine
Krankenhaushygiene	Hospital hygiene
Magnetresonanztomographie	Magnetic resonance imaging
Manuelle Medizin	Manual medicine/osteopathic adjustment
Medikamentöse Tumortherapie	Medical tumor therapy
Medizinische Informatik	Medical computer science
Naturheilverfahren	Naturopathy
Notfallmedizin	Emergency medicine
Nuklearmedizinische Diagnostik für Radiologen	Nuclear medicine diagnostics for radiologists
Orthopädische Rheumatologie	Orthopaedic rheumatology
Palliativmedizin	Palliative medicine
Phlebologie	Phlebology
Physikalische Therapie	Physical therapy
Plastische und Ästhetische Operationen	Plastic and cosmetic surgery
Proktologie	Proctology
Psychoanalyse	Psychoanalysis
Psychotherapie	Psychotherapy
Rehabilitationswesen	Interdisciplinary coordination and patient guidance in rehabilitative medicine
Röntgendiagnostik für Nuklearmediziner	Diagnostic radiology in nuclear medicine
Schlafmedizin	Sleep medicine
Sexualmedizin	Sexual medicine
Sozialmedizin	Social medicine
Spezielle Kardiologie für Erwachsene mit angeborenen Herzfehlern (EMAH)	Special Cardiology for adults with congenital heart defects
Spezielle Kinder- und Jugend-Urologie	Special child and adolescent urology
Spezielle Orthopädische Chirurgie	Special orthopaedic surgery
Spezielle Schmerztherapie	Special pain therapy
Spezielle Unfallchirurgie	Special trauma surgery
Spezielle Viszeralchirurgie	Special visceral surgery
Sportmedizin	Sports medicine
Suchtmedizinische Grundversorgung	Primary care in addiction medicine
Transplantationsmedizin	Transplant medicine
Tropenmedizin	Tropical medicine
